# Neuralgia—Introduction of Medicated Fluid to the Nerve

**Published:** 1845-08

**Authors:** 


### PRACTICAL MEDICINE, &c.

*Neuralgia—Introduction of Medicated Fluid to the Nerve.*—By Mr. Rynd,—Reported by Mr. Richard Gregory.—Margaret Cox, ætat 59, of spare habit, was admitted into hospital, May 18, 1844, complaining of acute pain over the entire of left side of face particularly in the supra-orbital region, shooting into the eye, along the branches of the portio dura in the cheek, along the gums of both upper and lower jaw, much increased in this situation by shutting the mouth and pressing her teeth close together, and occasionally darting to the opposite side of the face, and to the top and back of her head. She states that about six years ago she fell from a wall, and in the act of falling, a stone struck her in the temple; that twelve months after this she was much exposed to cold, and one night was suddenly seized with the most agonizing pain in the situations above described. "She thought her eye was being torn out of her head," and her cheek from her face; it lasted about two hours, and then suddenly disappeared on taking a mouthful of ice. She had not had any return for three months, when it came back even worse than before, quite suddenly, one night, on going out of a warm room into the cold air. On this attack, she was seized with chilliness, shivering, and slight nausea; the left eye lachrymated profusely, and became red with pain; it went in darts through her whole head, face and mouth, and the paroxysm lasted for three weeks, during which time she never slept. She was bled and blistered, and took opium for it, but without relief. It continued coming at irregular intervals, but each time generally more intense in character, until at last, weary of existence, she came to Dublin for relief.

She had been salivated three times, and had been so much in the habit of taking laudanum, that latterly half a drachm, three times in the day, had no effect in lulling the pain, and was the quantity she commonly took. She was a miserable, sallow-complexioned looking creature, had been sleepless for months, and her face was furrowed with constant pain.

On the 3d of June, a solution of 15 grains of acetate of morphia, dissolved in one drachm of creosote, was introduced to the supra-orbital nerve, and along the course of the temporal, malar, and buccal nerves, by four punctures of an instrument made for the purpose. In the space of a minute all pain (except that caused by the operation, which was very slight,) had ceased, and she slept better that night than she had done for months. After the interval of a week she had slight return of pain in the gums of both upper and under jaw. The fluid was again introduced by two punctures made in the gum of each jaw, and the pain disappeared. After this the pain did not recur, and she was detained in hospital for some weeks, during which time her health improved, her sleep was restored, and she became quite a happy looking person. She left the hospital on the 1st of August in high spirits, and promised to return if she ever felt the slightest pain again. We conclude she continues well, for we have not heard from her since.

Case II.—R. Dolon, ætat. 28, a thin spare man, of middle stature, was admitted into hospital 9th September, 1844, and came under Mr. Rynd's care on the 10th of November, complaining of acute pain in the right hip, thigh and leg, to the sole of the foot, along the entire course of the sciatic nerve and its branches, but chiefly in the main trunk of the nerve. He is unable to sleep, from the pain, and quite unable to walk. He is much emaciated, and the muscles of the limb are attenuated and wasted. He has been ill for three years, during which time he has been almost always confined to bed. He has been frequently treated for the disease with calomel, to produce salivation, cupping, blistering, leeching, &c., all without any salutary effect. Exposure to cold and wet is assigned as the cause of the disease.

On the 13th of November the fluid was introduced, ten grs. acetate morphiæ to the drachm of creosote, one puncture behind the trochanter, and one half-way down the thigh. He was instantly relieved from pain, and walked steadily through the ward without any pain or difficulty; before, walking increased the pain. For about half an hour after the operation he felt uneasiness from the puncture.

16th. Says he is perfectly well in the thigh, and feels only a slight pain in the course of the anterior tibial nerve. The fluid was again introduced to-day to the seat of pain by two punctures; it disappeared as before.

29th. Says he is perfectly well; has walked every day since; has slight stiffness in the knee from previous want ot use.

Ordered: Camphorated oil to rub the knee with.

December 15th. Left hospital to-day, saying he felt perfectly free from all pain and uneasiness.

February 6th. He walked up to Dublin to-day (20 miles), and says that since the last operation, on the 16th November, he has never felt his old pain, and is perfectly well.—*Dublin Med. Press*, in *Bulletin of Med. Science.*

The success of the same treatment is corroborated by a letter, directed to the Dublin Med. Press, and signed Arthur Guinness. Dr. Guinness used, for the introduction of the medicine, "a common lancet armed with morphine, mixed in a little water, about the consistence of paste, and operated precisely as is done in vaccinating an infant." With this he made several small punctures along the course of the nerve affected. In two cases, which he recites, he did not use creosote with the morphine, yet his success was perfect. In a subsequent case of neuralgia in the foot and leg, he used creosote *without morphine*, and this time also with success. This would seem to us to indicate that the modus operandi was by counter-irritation, or otherwise we must believe that creosote, thus applied, is possessed of anodyne properties.

